# Mourners’ Dissatisfaction with Funerals May Influence Their Subsequent Medical/Welfare Expenses—A Nationwide Survey in Japan

**DOI:** 10.3390/ijerph19010486

**Published:** 2022-01-02

**Authors:** Carl B. Becker, Yozo Taniyama, Noriko Sasaki, Megumi Kondo-Arita, Shinya Yamada, Kayoko Yamamoto

**Affiliations:** 1 Policy Science Unit, School of Medicine, Kyoto University, Kyoto 606-8501, Japan; 2 Department of Religious Studies, Tohoku University, Sendai 980-8576, Japan; tanimjp@mac.com; 3 Department of Healthcare Economics and Quality Management, Graduate School of Medicine, Kyoto University, Kyoto 606-8501, Japan; sasaki.noriko.3z@kyoto-u.ac.jp; 4 Nakayama International Center, Osaka Medical and Pharmaceutical University, Osaka 569-8686, Japan; megumi.kondo@ompu.ac.jp; 5 National Museum of Japanese History, Sakura 285-8502, Japan; shinya@rekihaku.ac.jp; 6 Department of Nursing, Tenri Health Care University, Tenri 632-0018, Japan; k.yamamoto@tenriyorozu-u.ac.jp

**Keywords:** grief, bereavement, mental health, public health costs, prevention strategies

## Abstract

Japan’s super-aged mortality rate bereaves millions of people annually, threatening the mental health of the bereaved population. Previous research suggests that participation in satisfying funeral rituals can protect or improve the health of a bereaved population—but pandemic restrictions threaten traditional funeral assemblies. To determine how bereaved mourners’ mental health—and consequent dependence upon medical, pharmaceutical, or social services—are affected by funerals and the aspects of funerals most likely to cause satisfaction or dissatisfaction, we conducted an anonymous nationwide survey across Japan. In total, 1078 bereaved Japanese responded; we analyzed their responses by comparing the 106 citing funeral dissatisfaction with the 972 citing no dissatisfaction. The cohort showing greatest satisfaction with funerals tended to be older widows or parents who lost children; they showed greater grief but spent less on medical, pharmaceutical, or social services thereafter than the dissatisfied. Conversely, mourners with the greatest *dis*satisfaction toward their interactions with funeral directors and Buddhist priests tended to spend more on medical, pharmaceutical, or social services after bereavement. We conclude that training or education to improve priests’ and funeral directors’ interactions may reduce dissatisfaction with funerals, potentially reducing subsequent costs of medical, pharmaceutical, or social services for the rapidly growing population of bereaved Japanese.

## 1. Introduction

Between 2015 and 2030, one-sixth of the Japanese population will have died from natural causes [1]. Since most such elders have more than six descendants, and deaths typically affect six or more family and intimate partners, by 2030, almost the entire Japanese population will have been bereaved by a grandparent, parent, or spouse. Other countries’ studies suggest that some 6% to perhaps 20% of a bereaved population show significantly increased distress and depression [2,3]. Thus, the overarching question arises: when an aging society becomes bereaved, how will their grief responses affect their subsequent health and dependence upon medical and social services?

Before the social distance rules of the COVID-19 pandemic, most bereaved adults shared one other common experience: the funeral of their loved one(s). After medical specialists, funeral directors are among the first whom the bereaved encounter after their loss; both their personal interactions and the rituals that they facilitate surely influence the psychological emotions and social interconnectedness of the bereaved thereafter. We know that cognitive and emotional preparedness for bereavement may significantly improve the grief trajectory for this population [4]. Anecdotal evidence abounds for the cognitive and emotional value of funerals, yet the roles and effects of funerals on bereaved health and medical dependence are inadequately documented for the general population.

Population surveys on funeral effects appear to have begun in the 1970s [5]. Sanders’ 1970s Tampa Bereavement Project led to her development of a Grief Experience Inventory (GEI) [6]; she concluded her lifework with the now famous statement, “funeral rituals become the glue that holds the bereaved together” [7]. Drawing upon some 800 visits to older adult mourners, Kraeer cited more mental health issues in grief recovery for adults with no funeral services (34% grief complications) than for those who had experienced full funeral services (12% complications) [8]. As Rando put it [9], “whether or not funeral directors or their critics like it—and regardless of whether they have received formal training in crisis intervention and mental health service—funeral directors directly influence the grief of the survivors they serve.” Using Sanders’ Inventory, Gamino reported that grieving individuals who felt that the funeral was “comforting,” or actively participated in the planning of the ceremony, reported statistically significantly fewer grief symptoms on the GEI [10]. In Ireland, funeral directors perform roles “as carers of the deceased, as carers of the immediate family of the deceased, and as protectors of the reputation of families” [11].

To summarize previous research into the functions of funerals, we follow Hoy’s [12] framework “helping families and communities navigate the early process of grief”: (1.1) respectful viewing/disposal of the body, (1.2) gathering community support, (1.3) significant symbols/ritual action, (1.4) connection to cultural heritage—and add (1.5) interactions with funeral directors. After briefly reviewing the relevant literature, we introduce our all-Japan survey connecting funeral (dis)satisfaction with the medical and social service use of the bereaved population.

### 1.1. Respectful Viewing/Disposal of the Body

Gorer’s classic work with 565 widows and widowers found that those who had viewed the body at the funeral had fewer mental adjustment problems than those who had not [13]. Potential benefits of viewing the body include coming to accept the death, encouraging a formal closure or farewell, bidding farewell, and remembering the deceased as resting peacefully [14]. Children attending visitations reported significantly better outcomes than those who did not—and showed significantly fewer PTSD symptoms at 25 months after parental death [15]. In-depth interviews have consistently confirmed that viewing the body contributed to healing acute grief in subsequent years [16,17,18]. As Worden concluded: seeing the deceased appear to be resting peacefully becomes a comforting symbol for the healthy resolution of grief [19].

### 1.2. Gathering Community Support

Funerals represent significant social occasions facilitating community support for the bereaved [20]. Silverman and Worden reported that funerals enabled family to more readily accept comforting social support, even after they had forgotten the details of the funeral [21]; similarly, group rituals provide connectedness and continuity that enhance later bereavement outcomes better than solitary rituals [22]. For many, the funeral provides a mechanism “to openly release pain and sorrow in the safe haven of family and a supportive kinship network” [23]. The funeral also raises community awareness of the bereaved family’s needs for support that might otherwise remain hidden and ignored [24]. Chinese research recognizes the importance of family and friends’ support, beginning with funerals, to reduce PTSD and depression thereafter [25].

### 1.3. Symbolic Ritual

In the face of tragedy or grief, meaningful rituals can assuage or express what words cannot [26]. Rituals affirm the value and continuity of life; they can elevate the process of saying goodbye to a more sacred level [27]. Funeral rites provide structure that can bring closure or initiate role reorganization [28]. Even in secularized Holland, rituals prove more effective than psychological help-seeking for bereaved families [29]. However, the breakdown of multi-generational families leads many people to abbreviate public funerals in favor of private family rituals, recently exacerbated by COVID-19 restrictions on assembly [30].

Among a variety of sacred rituals, music, in particular, may enable mourners to accept or release complex emotions [31]. In some cultures, singing together produces therapeutic results [32]; in Japan, the Buddhist *Nembutsu* chant has become a powerful ritual, salutary for bereaved Japanese [33]. A Japanese cohort listening to Buddhist chants showed reduced salivary stress markers compared with a similarly bereaved control group without chanting [34]. Thus funeral rituals may demonstrably affect bereaved mental health.

### 1.4. Cultural Heritage/Continuing Bonds

Rites of passage such as funerals and subsequent memorials unite mourners in a shared cultural heritage. In much of East Asia, home altars display memorial plaques or tablets honoring the names or photos of the deceased, where the living regularly remember or commune mentally with the spirits of their departed ancestors [35]. In Japan, Buddhist “continuing bonds” rituals, such as monthly home services in which the temple priest reads sutras and prays for the spirit of the departed, allow widows to feel the presence of their lost loved ones, enfranchising and overcoming their grief [36]. Chinese widows talk to photos of their deceased; these strong “continuing bonds” at 4 months correlated with better adjustment at 18 months [37]. In Korea, too, connectedness with ancestors proves important to mourners’ physical and mental worlds [38]. Even in the UK, where most bereaved families no longer subscribe to formal religions, many hold strong beliefs about the continuing existence and relations with their loved ones [39].

### 1.5. Interaction with Funeral Directors

Outside family and medical staff, funeral directors are typically the first responders to the grief of the immediately bereaved family. As families prepare for and participate in funerals, morticians can gently help them to negotiate and come to terms with this universal challenge to mental stability [40]. Funeral directors can serve as comforting and resourceful grief counselors, enabling the bereaved to talk about their feelings, educating them on the universality of grief, and making follow-up calls to support and encourage their clients after the funeral [41].

Death poses the ultimate existential threat to the bereaved family’s sense of meaningfulness. By encouraging the family to discuss their positive memories about the departed loved ones [28], to verbalize their relationship with the deceased and convey the significance of those experiences [42], and to relate their feelings before and during the funeral ceremonies [43], funeral directors can help them re-establish a sense of meaning in their damaged universe. When the bereaved family faces an overwhelming sense of powerlessness, funeral directors can give them a space to reassert choice and control [44]; this choice and control appear essential for families to find funeral practices helpful and therapeutic [45].

In sum, funerals—and their participants’ experiences—affect mourners’ grief trajectories and mental health, probably including their reliance upon medical, pharmaceutical, and social services over the months or years after their loss. However, research showing the effects of funerals on mourners’ mental health is notably inconclusive; such research is particularly important to super-aging societies such as Japan, which pay for most medical and welfare expenses from public coffers. Our research was an attempt to shed some light on these correlations in Japan.

We wanted to determine whether funeral satisfaction or dissatisfaction would impact a recently bereaved population’s mental health and medical and social service use. However, as in the Bath/Dignity survey of cremation satisfaction in England [46], when the vast majority of respondents are “quite satisfied” to “very satisfied,” it is difficult to connect differences in health outcomes to funeral satisfaction [47]. Therefore, we formulated the primary hypothesis that funeral dissatisfaction would lead to greater dependence upon medical and social services within a year after bereavement. In the process, we wanted to know what factors within the funeral (such as viewing, rituals, meals, and interaction with priests/funeral directors) were most responsible for dissatisfaction. We also wanted to find the differences between those who express dissatisfaction with the funerals for their loved ones and those who did not, and which cohorts of the population (in terms of age, employment, relation to the deceased, etc.) were most likely to report funeral dissatisfaction.

## 2. Materials and Methods

### 2.1. Survey Procedure

From 2018 through 2021, the Japanese Ministry of Education, Science, and Technology (MEXT) funded a nationwide team of professional academic researchers to explore and examine the above factors in Japanese society. The researchers repeatedly gathered at Kyoto University to discuss the information they should seek and the questions they should ask of the bereaved. They considered the work of many previous surveys, taking special note of the Bath/Dignity survey on direct cremation [46]. Although Japan’s national social insurance system provides medical coverage for its population, hospitals and medical services neither follow up nor maintain contact records on the medical trajectory of bereaved families. In order to contact bereaved families, we relied on the services of funeral organizations and Buddhist priests (who typically preside at services and inter the ashes of the deceased).

In 2018, temple priests distributed pilot questionnaires to 250 chief mourners, from whom we received 170 complete responses. Their comments enabled us to improve the wording of the questions, reducing an eight-page tome to five pages with a clearer and simpler format. In 2019, the All-Japan Funeral Cooperative^®^ distributed more than 5500 of these revised questionnaires to families they had served in the past year, from which 1087 perfectly completed responses were received in 2020. The completed questionnaires were forwarded unopened to a professional data agency that anonymized and input the data, analyzed using IBM SPSS Statistics version 23. The following analyzes and discusses those results, especially from the perspective of funerals and rituals.

### 2.2. Survey Content and Theory

The survey began by collecting data about the age, education, and income of the respondents, followed by questions about their relationship to the deceased, the cause(s) and location of death, and their religious views and practices. To evaluate bereaved respondents’ psychological grief, we adopted the widely used Japanese translation of Prigerson’s PGD-13 scale [48] (with grateful permission), which uses items such as “In the past month, how often have you had intense feelings of emotional pain, sorrow, or pangs of grief related to the lost relationship?” Its answers range from (1) not at all to (5) constantly/overwhelmingly on a 5-point Likert scale, with higher scores indicating higher psychological grief. This PGD-13 scale has shown high internal consistency (Cronbach’s alpha ranging from 0.82 to 0.93) [49], and its translation has been widely used in Japan (Cronbach’s alpha of 0.92) [50,51].

To try to grasp their physical symptoms of grief, we used the Japanese translation of Pfizer’s Patient Health Questionnaire (PHQ-9J) Screener [52], including nine items such as “I can’t sleep, or I’m sleeping way too much,” “I have no appetite, or I’m eating excessively,” with answers ranging from (1) never to (4) almost daily, on a 4-point Likert scale, where higher scores indicate greater somatic symptoms. Pfizer’s Japanese PHQ-9 also shows high internal consistency (Cronbach’s alpha 0.93) [53]; detailed RMSEA/CFI analysis has been substantiated [54,55]. Both the PGD-13 and the PHQ-9 have been commonly adopted and used in Japan.

Several researchers suggest the effects of financial burden on bereavement grief [56]. Therefore, we too asked our respondents not only about income but also about how burdensome they felt their funeral expenses and their subsequent medical/social expenses, to compare feelings of financial burden with employment and income levels. Since pharmaceutical use was particularly conspicuous in the pilot survey, we divided our full survey items into analgesics (medicines for physical pain) and psychotropics (tranquilizers, antidepressants, etc., for mental issues). We asked about the cost and frequency of the above services over the past month (within a year after bereavement) and the increase or decrease in their use compared with pre-bereavement levels.

To understand mourners’ satisfaction or dissatisfaction with funerals, we listed items most typical of a Japanese funeral in the order of their typical performance, beginning with the family’s interactions with the funeral director/mortuary and their purification of the corpse, through items such as altar arrangements, Buddhist priests’ participation, the traditional memorial banquet, and the dividing of the ashes after cremation (Table 1). In terms of Hoy’s categories, our questions 1 and 3 covered Hoy’s (1.1) respectful viewing/disposal of the body; question 9 connects to (1.2) gathering community support; 4, 5, 6, and 7 relate to Hoy’s (1.3) significant symbols/ritual action and (1.4) connection to cultural heritage; and questions 2 and 8 to (1.5) interactions with funeral directors and priests. The respondents ranked these on a four-point scale of “disappointing,” “somewhat dissatisfied,” “as expected,” and “exceeded expectations,” where a higher score indicated higher satisfaction. We grouped those indicating any dissatisfaction into a “dissatisfied” cohort, and those indicating no dissatisfaction into a “satisfied” cohort.

Repeated screening of the pilot showed no significant differences or trends between those with moderate to high satisfaction with the funeral of their loved one. This is analogous to the inability of the Bath/Dignity survey to find significant differences between moderately satisfied and highly satisfied funeral celebrants [47]. By contrast, our pilot sample showed significant differences between the minority with mild to strong dissatisfaction and the majority with moderate to high satisfaction even after Bonferroni correction. This makes sense because it is implausible that funeral satisfaction alone should improve health after bereavement, but it is conceivable that funeral dissatisfaction might correlate with lingering regrets or anger, sleeplessness, psychosomatic aches and pains, and thus with subsequent doctor/hospital appointments. To test this dissatisfaction hypothesis, we used an inclusion criterion grouping all those who expressed mild to strong dissatisfaction with one or more elements of the funeral into a “dissatisfied” minority (*n* = 106) to compare them with the majority (*n* = 972) who expressed moderate to high satisfaction with all elements of the funeral.

### 2.3. Analyses

Data preparation and preliminary analysis were conducted using SPSS (Knowledge Data Service, Kyoto, Japan). Data were screened for normality, outliers, and missing response categories. Categorical variables were summarized into numbers and percentages. To conduct Welch *t*-tests with an effect size of 0.5 at ≤ 0.05 significance and a power of 0.9, we anticipated a need for at least 86 in each category; thus, with more than a thousand responses, we expected our response numbers should suffice for comparisons in each category. We compared “dissatisfied” and “not dissatisfied” using a series of bivariate Welch’s *t*-test correlations. Since this was an exploratory study, the researchers chose to leave α at 0.05 to allow any differences to be evident. The potential for type one errors without Bonferroni corrections remained, but given the investigative nature of this particular study, we thought such a move might conceal interesting tendencies; thus, we present the data in its present form without Bonferroni correction or multivariate comparative analysis at this stage.

## 3. Results

Beginning with funeral satisfaction/dissatisfaction (Table 1), the line item showing the lowest mean average satisfaction was interactions with Buddhist priests in rituals during and after the funeral; however, this was due to the fact that fewer satisfied mourners ranked their interactions “better than expected,” rather than to widespread dissatisfaction.

The category with the greatest difference—the worst rating in the dissatisfied cohort and a high rate of exceeding expectations in the satisfied cohort—was “discussion and interaction with the funeral director.” Interactions with funeral directors gathered the strongest negative reactions among the dissatisfied but showed high ranks among the satisfied; this great gap between satisfied and dissatisfied suggests that funeral directors leave a strong impression and great impact on bereaved satisfaction with funerals.

Curiously, the next most-criticized item (by both satisfied and dissatisfied groups) was the funeral banquet—but from numerical responses alone, we are unable to determine how much of this was due to the menu and catering service or how much due to the nature of the interactions between family, friends, and relatives. Conversely, the least dissatisfaction was voiced toward the number of people who attended—perhaps because this was the category in which mourners most personally participated in choosing and providing the funeral director with addresses and contact information—so the idea that more celebrants would provide more subsequent social support cannot be deduced from this data. The altar, decorations, and rituals of placing flowers in the coffin also showed relatively high satisfaction even among the “dissatisfied” group. Each of these elements of funerals contributed significantly to the satisfaction or dissatisfaction of the bereaved, but none of them individually connected to subsequent mental health as observed in medical or social service use.

The details of age, family, education, income, relationships, and other socio-economic factors are too massive—and too insignificant—to list in their entirety. For purposes of reporting here, we introduce only those categories in which the *t*-test differences between the bereaved satisfied and dissatisfied with their funerals approached statistical significance (Table 2).

The bereaved over the age of 70 showed significantly higher satisfaction with their loved ones’ funerals, whereas, for those under 70, there was no significant difference in satisfied and dissatisfied groups. Those who had a regular religious practice commemorating or memorializing the deceased were slightly more likely to express satisfaction with funerals than those who did not, but reported level of religious faith and belief in after-life showed no significant correlation. Regardless of bereavement age, the most dissatisfaction was shown toward grandparents’ funerals, and the greatest satisfaction toward funerals for spouses or children. Those bereaved in intensive care units (ICUs) were more likely to show dissatisfaction than those bereaved in all other locales.

Funeral satisfaction showed no statistically significant correlation to total income level, except for a very slight tendency to report dissatisfaction among those in lower-income brackets (under 4 million yen annually). However, when children were the primary payers for the funeral, their dissatisfaction significantly exceeded the cases when spouses or parents paid for the funeral.

On the Pfizer Patient Health Q9 Scale (Table 3), sleep disorders were somewhat conspicuous among all mourners, but significantly worse among those dissatisfied with their funerals. Loss of interest in life, depression/hopelessness, and lethargy or constant fidgeting (behavioral disorders) were less common in total, but appeared as tendencies bordering on significance among those dissatisfied with funerals. Wish to injure or kill oneself was fortunately minimal across our sample, but again noticeably higher among the dissatisfied cohort, although statistical significance could not be shown for this minority. Funeral dissatisfaction did not seem to affect physical pain as much as it did subsequent psychological and mental health complaints.

About a third of our 1078 bereaved respondents reported using medical, pharmaceutical, or welfare services; one-third of that number (11%) reported significant increases in service expense after bereavement (Table 4). 

The percentage of those satisfied and dissatisfied with funerals using medical, pharmaceutical, or welfare services showed no statistically significant difference; in other words, the number of bereaved and their frequency (in visits per month) that tended to turn to doctors, to pharmacies, or to financial, legal, and/or welfare advisers had no relation to their funeral satisfaction. However, the minority who were dissatisfied with funerals paid thrice as much per visit for doctor/hospital appointments and four times as much per visit for financial, legal, and/or welfare advisers as those satisfied with funerals. These visits were not made more frequently, but the cost of each visit mushroomed, suggesting that they required more detailed medical examinations, more tranquilizers or sleeping medicines, and longer financial or welfare consultations. Finally, those who considered the funeral to be a significant or debilitating expense tended to increase their use of medical/social services after the funeral more than those who felt that expense to be a moderate or negligible burden (Table 5). 

## 4. Discussion

Our results concur with earlier studies [57], showing that those reporting a very close relationship to the deceased tended to value all aspects of funerals more positively, and that older adults were typically more appreciative of traditional funerals than were younger adults [58]. Similar to O’Rourke [59], we found that religious practice correlated with higher funeral satisfaction but not with less grief, analogous to the tendency for elders and widows to show greater grief and less funeral dissatisfaction. Our results also confirm that predetermined funeral plans avoided dissatisfaction better than suddenly facing the need to both decide on and pay for a parent’s or grandparent’s funeral [60].

Our respondents’ rankings of satisfaction with elements of the funeral showed no significant correspondence to mental health, grief profiles, or frequency of visiting medical and social services. Our survey does not show that funeral satisfaction buffers mourners’ grief; it focuses rather on funeral dissatisfaction and service use. Subjectively, the *dis*satisfied cohort reported more sleep disorders and more tendencies to depression, lethargy, or loss of interest in life than the satisfied cohort. Interestingly, this sleep-disturbed and depressed cohort was more likely not to be senior citizens but to be children or grandchildren of the deceased. While this did not change the frequency of their visits to doctors, hospitals, pharmacies, or welfare/financial advisers, it appears to have substantially affected the amount they spent on these services, compared with those who used the same services but were quite satisfied with their funerals.

The most significant causes of such dissatisfaction with funerals were the words, attitudes, and interactions of their presiding priests and funeral directors. Apparently, this left unpleasant or regrettable memories with the bereaved, perhaps affecting their sleep or even will to live. While the vast majority of funerals in Japan (as in the UK) met or exceeded their bereaved celebrants’ expectations, the minority of funerals that betrayed, disturbed, or offended those hopes may be partly responsible for additional mental health issues lasting months thereafter. Contrary to the indications of previous research [34], both satisfied and dissatisfied mourners appraised their interactions with Buddhist priests as another of the least satisfying aspects of bereavement ritual. This recognition of the potential influence of priests and funeral directors might suggest creating programs for improving their training in interpersonal interaction with the suddenly bereaved, to contribute to their mental health and even to reduce national medical/welfare expenses.

Our findings that some 4% to 20% of bereaved depended upon medical and social services (16–20% medical/hospital appointments, 12–14% analgesics, 4% psychotropics, 13–14% welfare appointments) are within the range we might expect to report continuing needs for support [2]. The finding that funeral satisfaction/dissatisfaction did not significantly affect the frequencies or percentages of people relying on these medical and social services, but that dissatisfied bereaved tended to spend significantly more money on each of their medical visits, medicines, and financial/welfare services is especially interesting. Statistically, those dissatisfied were unlikely to be older adults or lonely widows whom we might expect to have more medical, physical, or financial/welfare problems. Widows who had lost husbands and parents who had lost children expressed somewhat greater psychological grief, but at the same time, greater satisfaction with the funerals. By contrast, it was children or grandchildren of the deceased who showed more dissatisfaction with funerals and tended to find funeral costs burdensome, but paid more for their own medical appointments, drugs, and financial/legal/welfare services. They tended to be in their 40s to 60s, less religious, and/or more responsible for paying for the funeral. We might expect them to be healthier than their surviving older parents or grandparents, but their higher expenditures on medical and welfare services suggest this was not necessarily the case.

Funeral dissatisfaction seems significantly connected to increased personal expenses for medical and social services. Currently, the Japanese government typically pays 70–80% of such expenses, meaning that for every dissatisfied respondent who paid 11 thousand yen (US$100) more than did satisfied respondents, the government (taxpayers) probably paid 26 to 44 thousand yen (US$240–400) more per month. Even if this group (whose bereavement and dissatisfaction caused them to pay higher medical and welfare fees) were as few as 1% of the entire bereaved population, that might still constitute 90,000 people, costing the government about US $300 million per year (even assuming that previous years’ bereaved costs never continue into future years).

## 5. Limitations

To be sure, we can draw no absolute causal connection from funeral dissatisfaction to higher medical and welfare expenses. It is just possible that other common factors underlie these mourners’ dissatisfaction with funerals and their tendencies to spend more on medical and welfare issues. Possibly some underlying malaise, grief, or psychological instability was triggered or aggravated by their dissatisfying interactions with the funeral directors. 

The number of people attending the funeral was the category that showed the least significant difference between satisfied and dissatisfied groups—but we lack data for funerals since COVID-19 restrictions; so we have inadequate grounds to discuss the effects of COVID-19 restrictions on funeral dissatisfaction.

The many variables in this survey call out for more rigorous structural equation modeling with statistical corrections for multiple variables; this report is a mere introduction of the rich data gathered to date. We are in the process of reading and analyzing thousands of free-response items and marginal comments to further clarify the logic behind these correlations and contacting those who agreed to respond for further follow-up analysis.

## 6. Conclusions

A loved one’s funeral can leave deep memories that impact the mental health of the bereaved over the following months and years. Previous research suggests that funerals might buffer or improve this grief trajectory, but pandemic restrictions on public assemblies further threaten the value or utility of funerals to the bereaved. The least we can do is to try to reduce funeral dissatisfaction that may adversely affect the mental health and welfare of the bereaved. Our nationwide survey of bereaved in Japan sought to identify the reasons for satisfaction or dissatisfaction with funerals and to examine whether that connected to other aspects of health and welfare dependence.

Satisfaction with funerals was highest among elders; while funeral satisfaction did not clearly buffer their grief at losing spouses or children, they did not spend noticeably more on health or welfare after bereavement than prior to it. Funeral dissatisfaction was highest among those losing grandparents, or in ICUs, or suddenly burdened with paying for funerals. Those bereaved who were dissatisfied with funerals visited medical, pharmaceutical, and welfare service providers at the same frequency as those who were satisfied with funerals but tended to spend 3–4 times more money per visit than the satisfied cohort. Since the Japanese government substantially subsidizes most of these expenses, such funeral dissatisfaction may be a cause for public concern. Prior to the COVID-19 pandemic, satisfaction was highest with the number of people who attended—perhaps because this was actively negotiated by the bereaved. When COVID-19 prohibits large funeral gatherings, this may add a new source of dissatisfaction, with further psychosocial ramifications. In our pre-COVID-19 sample, dissatisfaction tended to concentrate on the mourners’ interactions with funeral directors and Buddhist priests. Since funeral directors and priests are among the first whom the bereaved encounter after their loss, their influence looms particularly strong. Members of our research team are working with Japan’s fledgling Buddhist chaplain training program and with the All-Japan Funeral Cooperative^®^ to improve and maintain higher professional competence in their interactions with the recently bereaved who seek their services. Better training of funeral directors’ and priests’ psychosocial skills to avoid leaving dissatisfied memories might reduce mourners’ subsequent expenditures on medical, pharmaceutical, or social services.

## Figures and Tables

**Table 1:** Evaluation of elements of the funeral (in order of ritual performance, not functionality).

Evaluations of Elements of the Funeral (Excluding Unused) (=Difference in Mean Satisfaction between Cohorts)	Average	Dissatisfied (106)	Satisfied (972)	*p*
1 Last rites, purifying and dressing the deceased (0.79)	3.25	2.54	3.33	0.001 ***
2 Discussion and interaction with the funeral director (0.95)	3.35	2.49	3.44	0.001 ***
3 Moving the body; placing flowers/possessions in the coffin (0.71)	3.37	2.73	3.44	0.001 ***
4 The altar and surrounding flowers/decorations (0.67)	3.43	2.83	3.50	0.001 ***
5 The religious ceremony at the funeral (0.73)	3.25	2.60	3.33	0.001 ***
6 The ceremonial banquet (0.76)	3.22	2.53	3.29	0.001 ***
7 The crematorium ceremonies (dividing the ashes) (0.68)	3.26	2.65	3.33	0.001 ***
8 Subsequent interactions with or memorial services by the priest (0.65)	3.16	2.57	3.22	0.001 ***
9 The number of people who attended (0.43)	3.29	2.90	3.33	0.001 ***

*p*-value shows significant difference between dissatisfied and satisfied groups’ mean scores; *** = *p* ≤ 0.001.

**Table 2:** Socioeconomic variables connected to funeral satisfaction/dissatisfaction.

Respondents (*n*)	Total (1078)	Dissatisfied (106)	Satisfied (972)	*p*
**Respondent’s Age**				
70’s	12.2%	3.8%	13.1%	0.01 **
80’s or 90’s	3.5%	0.0%	3.9%	0.07
**Regular religious practice towards the deceased**	53.4%	43.4%	54.5%	0.04 *
**Deceased’s relation to the bereaved**				
Grandparent	16.4%	28.3%	15.1%	0.001 ***
Father	27.1%	33.0%	26.4%	0.18
Spouse or child	16.6%	7.5%	17.6%	0.01 **
**Location of Death**				
ICU	12.6%	19.8%	11.8%	0.03 *
**Employment (%)**				
Employed Full Time	50.6%	64.2%	49.1%	0.001 ***
Not Working	20.9%	10.4%	22.0%	0.01 **
Income < 4 million yen	28.8%	35.8%	28.0%	0.11
**Primary Funeral Payer**				
The deceased’s Child	37.0%	48.1%	35.8%	0.02 *
Deceased’s Spouse and Child together	5.8%	0.9%	6.4%	0.04 *

Topic categories shown in bold; subgroups showing noticeable differences are non-bold. *p*-value shows significance of difference between dissatisfied and satisfied mean scores. * = *p* ≤ 0.05, ** = *p* ≤ 0.01, *** = *p* ≤ 0.001.

**Table 3:** Public Health Scale differences between dissatisfied and satisfied groups.

Pfizer PHQ-9 Ratings	Total (1078)	Dissatisfied (106)	Satisfied (972)	*p*
Lost interest in everything	1.42	1.53	1.40	0.10
Depressed, hopeless	1.44	1.55	1.42	0.08
Sleep disorders	1.56	1.73	1.54	0.05 *
Lethargic or fidgety	1.21	1.30	1.20	0.08
Self-injury or death wish	1.10	1.18	1.10	0.14

Higher numbers show higher incidence of public health problems. *p* shows difference between dissatisfied and satisfied groups’ public health scores; * = *p* ≤ 0.05.

**Table 4:** Expense comparison of bereaved dissatisfied and satisfied with funerals.

Amount You Paid in the Past Month for:	Average Spent by All Users [User% of Total]	Average Spent by Dissatisfied [*n* = 106]	Average Spent by Satisfied [*n* = 913]
Medical/hospital appointments	7410.3 [19%]	17,570.6 [16%]	6486.6 [20%]
Analgesic Medicines (headaches, stomach aches, backaches, cramps, etc.)	2154.3 [12%]	2546.7 [15%]	2101.8 [12%]
Psychotropic Medicines (tranquillizers, antidepressants, sleep medicines, etc.)	1016.3 [4%]	2500.0 [4%]	864.1 [4%]
Psychiatric/Psychological counseling (face to face)	843.8 [1.5%]	1000.0 [1%]	833.3 [1.6%]
Financial, legal, or welfare advisors	32,254.2 [13%]	104,707.7 [13%]	25,064.1 [14%]

Costs in Japanese Yen [percent of users in brackets; no significant difference in percent]; No statistical difference in percentage of users, but noteworthy differences in mean average expenses.

**Table 5:** Relation of funeral’s economic burden to increased medical/social service use.

Economic Burden of Funeral	Total (1078)	Increased Use (122)	No Increased Use (956)	*p*
Felt No Burden	37.2%	29.5%	38.2%	0.08
Somewhat of a Burden	47.1%	50.8%	46.7%	0.44
Significant Burden	10.2%	15.6%	9.5%	0.05 *
Debilitating Burden	2.0%	3.3%	1.9%	0.44

*p*-value shows significance of difference of increased and non-increased users’ scores; * = *p* ≤ 0.05.

## Data Availability

Data are not yet made publicly available due to further analysis.
